# A multidisciplinary staff for the optimisation of therapy in HIV-infected patients treated for cancer

**DOI:** 10.1186/1742-4690-9-S1-P66

**Published:** 2012-05-25

**Authors:** Julie Daigre, Meriem Mendjel, Sylvie Bregigeon, Catherine Tamalet

**Affiliations:** 1 APHM La Timone, Laboratoire de Pharmacocinétique et Toxicologie, Marseille, France; 2 APHM Sainte Marguerite, Service d’Immunohématologie clinique, CISIH, Marseille, France; 3 Pole des Maladies Infectieuses et tropicales Clinique et Biologique, Fédération de Bactériologie Hygiène Virologie, Marseille, France

## Introduction

With the improvement of HAART, life expectancy of HIV-infected patients sharply increased. The incidence of cancerous diseases is therefore increasing in this population. This raises the problem of drug-drug interactions between HIV treatment and cancer chemotherapy poorly understood because few data are available. However, the potential risk of interactions is important because of the involvement of similar metabolic enzymes and transporters between these drugs and due to the inhibitory and/or inducer effects on different CYP450 isoforms of the PI and NNRTI. These interactions may both lead to inefficiency and/or an increased risk of toxicity of the chemotherapy which can be life-threatening for the patient.

## Materials and methods

We set up since January 2010 amultidisciplinary staff “AIDS-cancer-transplantation” to optimize the management of HIV-infected patients diagnosed for a tumour disease. Demographic, virological and immunological characteristics and therapeutic decision issued from the staff for these patients have been retrospectively analyzed.

## Results

As of today, data of 28 patients have been evaluated. A therapeutic adjustment had to be proposed for 14 patients (50%), mainly corresponding to a modification of the antiretroviral therapy (11/14). The new cART therapeutic option was defined according to HIV resistance profile, ART history and after additional biological analyzes if requested. In few cases (4/14), the cancer chemotherapy was adjusted according to the different therapeutic options proposed. The main reasons for such therapeutic adjustment were, for 57% (8/14), related to a metabolic interaction through the CYP3A4 because of the presence of a boosted PI or NNRTI and for 36% (5/14) to an increased risk of nephrotoxicity due to the concomitant administration of tenofovir with a potent nephrotoxic anticancer drug.

For all patients, opportunistic infections prophylaxis (PCP) and screening for PCR CMV have been prescribed according to French recommendations.

## Conclusion

The implementation of the multidisciplinary staff “AIDS-cancer-transplantation” highlights a high frequency of the risk of drug interactions between antiretrovirals and antitumoral agents. This allows us to optimize the management of HIV patients treated for malignant diseases by apprehending the risk of drug interactions.

